# Supplementary material for: Close to open—Factors that hinder and promote open science in ecology research and education
Source: PLoS One. 2022 Dec 21;17(12):e0278339. doi: 10.1371/journal.pone.0278339 (PMC9770360; doi:10.1371/journal.pone.0278339)
Supplement: S1 File — (PDF) [file pone.0278339.s001.pdf]

CLOSE TO OPEN - FACTORS THAT HINDER AND PROMOTE OPEN SCIENCE IN ECOLOGY RESEARCH AND EDUCATION

Supporting Information

Table 1: Program for the Living Norway 2020 Colloquium

Day	Time	Activity	Description	Collaboration with
I		Session 1	Living Norway & the biodiversity informatics landscape	
	10:00-12:00	Welcome: Norunn Myklebust, Managing director NINA<br>Living Norway Ecological Data Network - Status and vision: Erlend B. Nilsen, Senior research scientist, NINA<br>The why, how and when to use data standards in ecology: Anders G. Finstad, Professor, NTNU University Museum<br>Improved data management with LivingNorwayR: Matt Grainger, Postdoctoral fellow, NINA		
		Panel discussion		
	12:00-13:00	Lunch		
		Session 2	Open science in basic and applied ecology and beyond	
	13:00-14:15	Open science in general: Ingrid Heggland, Senior research librarian, NTNU Library<br>The status of the â€œreproducibility crisisâ€ in the wildlife sciences: Althea Archer, Assistant Professor, St. Cloud State University and John Fieberg, Associate Professor of Quantitative Ecology, University of Minnesota		
		How can journals support open data in ecology? Emilie AimÃ©, Managing Editor, British Ecological Society<br>Panel discussion		
	14:15-14:45	Coffee break		
		Session 3	Open science and fair data in the science-policy interface	
	14:45-16:00	IPBES goes FAIR! Lessons Learned and the Way Forward: Rainer Krug, Lead of data management working group of the IPBES task force on knowledge and data. PhD, University of ZÃ¼rich		
		Panel discussion		
II				
	09:00-12:00	Workshop	Education and training in open science and FAIR data management.	SFU bioCEED
	12:00-13:00	Lunch		
	13:00-16:00	Workshop	Statistical modelling of new open data sources. We will, in particular, discuss models that integrate information from a range of different data sources simultaneously.	SFF Centre for Biodiversity Dynamics, CBD

Table 2: Survey questions distributed among attendees at the Living Norway 2020 Colloquium

Part	Question	Options	Response
I	People define 'Open Science' in many ways, and it's a multi-faceted concept. We are interested in how you define Open Science, especially as it pertains to your own work. Please take a minute to share these thoughts. The more detailed, the better.		Open-ended
	What Open Science activities have you engaged in?	Shared data openly<br>Shared code openly<br>Used open data<br>Used open code	Never, Rarely,<br>Several times a year,<br>Several times a month,<br>Several times a week,
		Published my papers openly<br>Used open education tools or practices<br>Read open papers<br>Engaged in open peer review<br>Engaged in outreach/Science communication	I don't know
		Other (please specify)	
	What has hindered you from engaging in Open Science?		Open-ended
	What has helped you engage in Open Science?		Open-ended
	How important are the following aspects of Open Science to your RESEARCH?		
		Data sharing, Code sharing	Not applicable to my work,
		Scientific Publication	Minimally important,<br>Somewhat important,
		Outreach	Very important, Extremely important
		Research Reproducibility<br>Research Transparency	
	How important are the following aspects of Open Science to your TEACHING?		
		Data sharing, Code sharing<br>Scientific Publication	Not applicable to my work,<br>Minimally important,<br>Somewhat important,
		Outreach	Very important, Extremely important
		Research Reproducibility<br>Research Transparency	
	How important are the following aspects of Open Science to your SUPERVISION (of graduate students and postdoc)?		
		Data sharing, Code sharing<br>Scientific Publication	Not applicable to my work,<br>Minimally important,<br>Somewhat important,
		Outreach	Very important, Extremely important
		Research Reproducibility	
		Research Transparency	
	Please feel free to explain your answers to any of the above.		Open-ended
	What is your current affiliation type? Please select all that apply.	University, Research institute, Governmental agency, Private company, Other	Constrained choice
	In which country do you work or study? Please select all that apply.	Norwegian, EU/EEC, non-EU/EEC	Constrained choice
	What is your current position?	Bachelor student, Master student, PhD-student, Researcher (temporary, researcher (permanent), Associate professor/Professor, Other	Constrained choice
	What is your highest degree?	BSc, MSc, PhD	Constrained choice
	When did you finish your highest degree?		Open ended
	Are you involved in any of the following types of teaching? Please select all that apply.	Undergraduate classes, Doctoral or master classes, Supervising undergraduates, Supervising doctoral or master students, Supervising postdoctoral scholars, Public outreach, Other	Constrained choice
	What is your gender?		Open-ended
	What parts of this workshop are you attending	Day one, Education workshop, Analysis workshop	Constrained choice
	How are you attending the workshop?	Physical, Virtual, Both	Constrained choice

Table 3: Survey questions distributed among attendees at the Living Norway 2020 Colloquium

Part	Question	Options	Response
II	Do you teach?	Yes, No	Constrained choice
	Do you supervise MSc-students, PhD-Students and/or postdoctoral researchers?	Yes, No	Constrained choice
	Have you encountered open science practices in your personal education experience?	Yes, No, Don't know	Constrained choice
	If yes (or donâ€™t know), which?		Tick-boxes
		Read open-access literature / material (e.g. papers, textbooks)	
		Used open data	
		Used open code	
		Shared own data openly	
		Shared own code openly	
		Published results or papers openly	
		Been taught principles of research transparency	
		Been taught principles of research reproducibility	
		Open peer review	
		Outreach/Science communication	
		Other (please specify)	
III	People define 'Open Science' in many ways, and it is a multi-faceted concept. We are interested in how you define Open Science, especially as it pertains to your own work. Please take a minute to share these thoughts. The more detailed, the better.		Open-ended

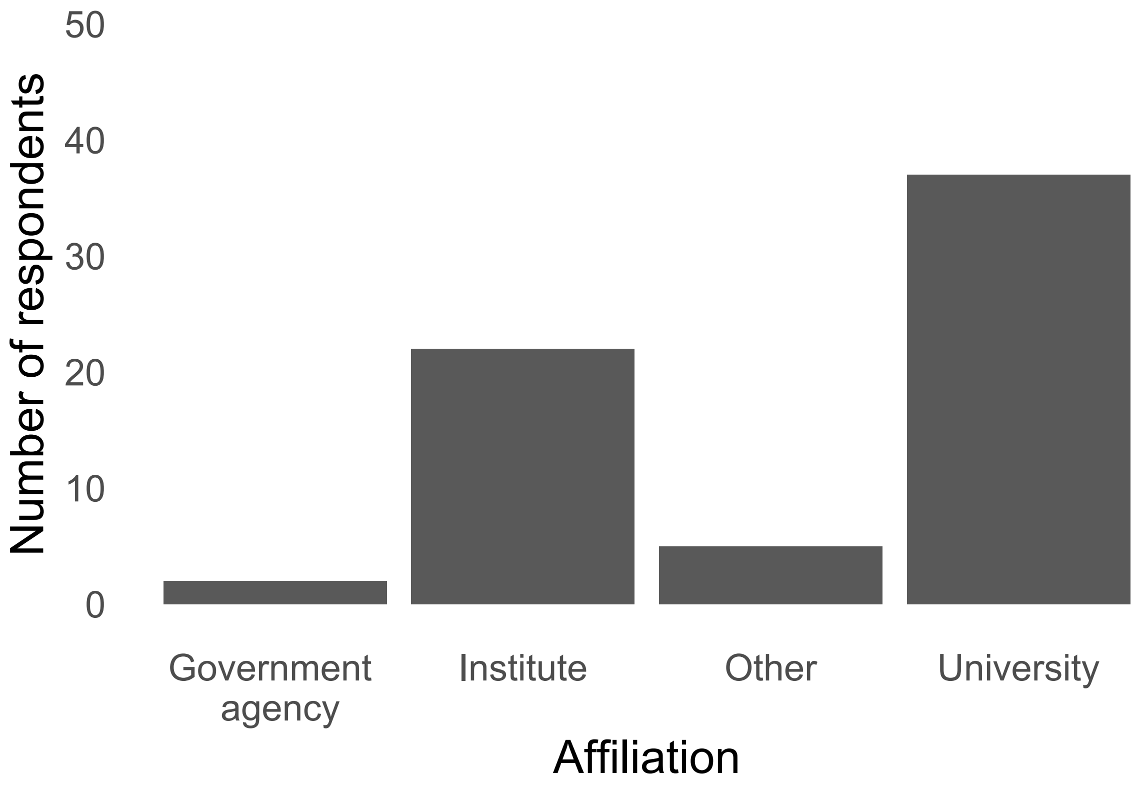

Figure 1: Number of respondents by affiliation.

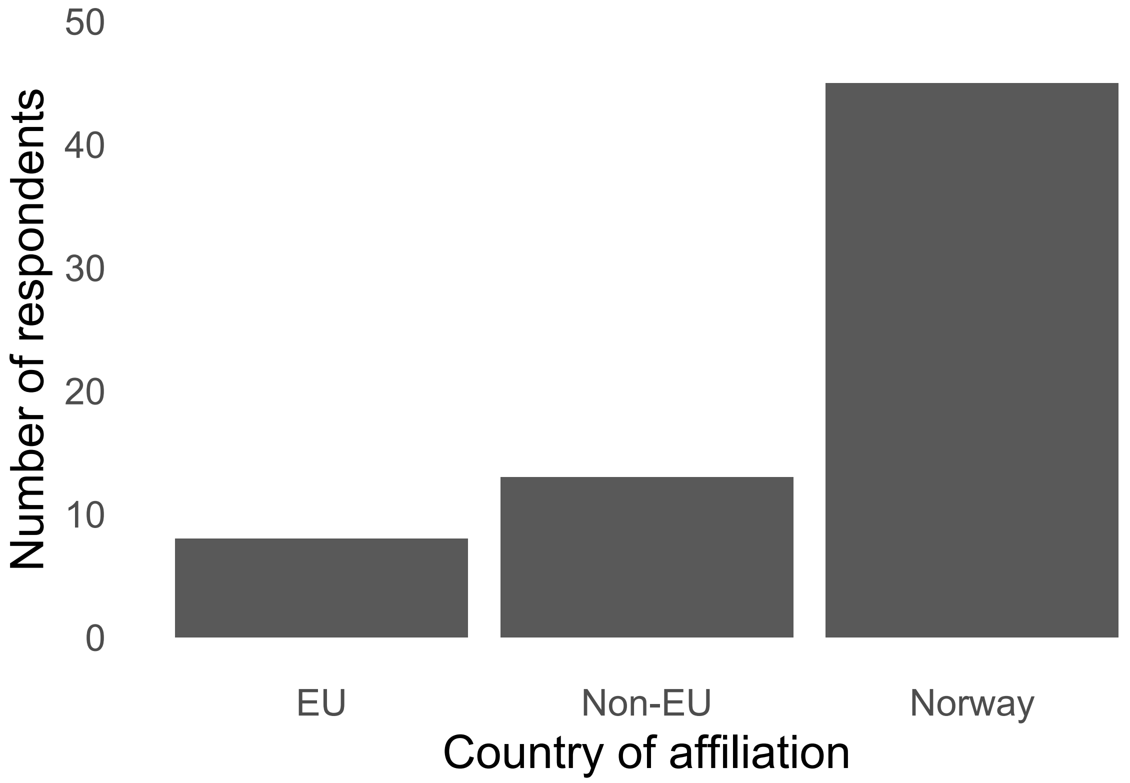

Figure 2: Number of respondents by contry.

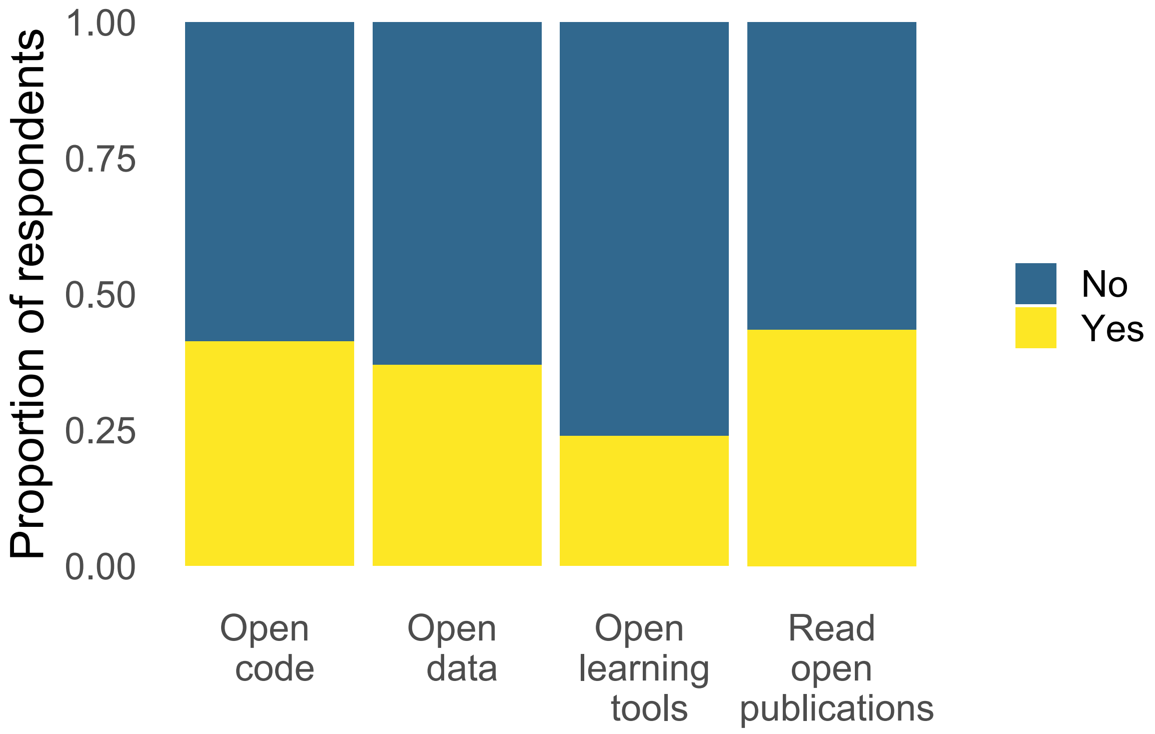

Figure 3: Respondents that had used open resources in own education.

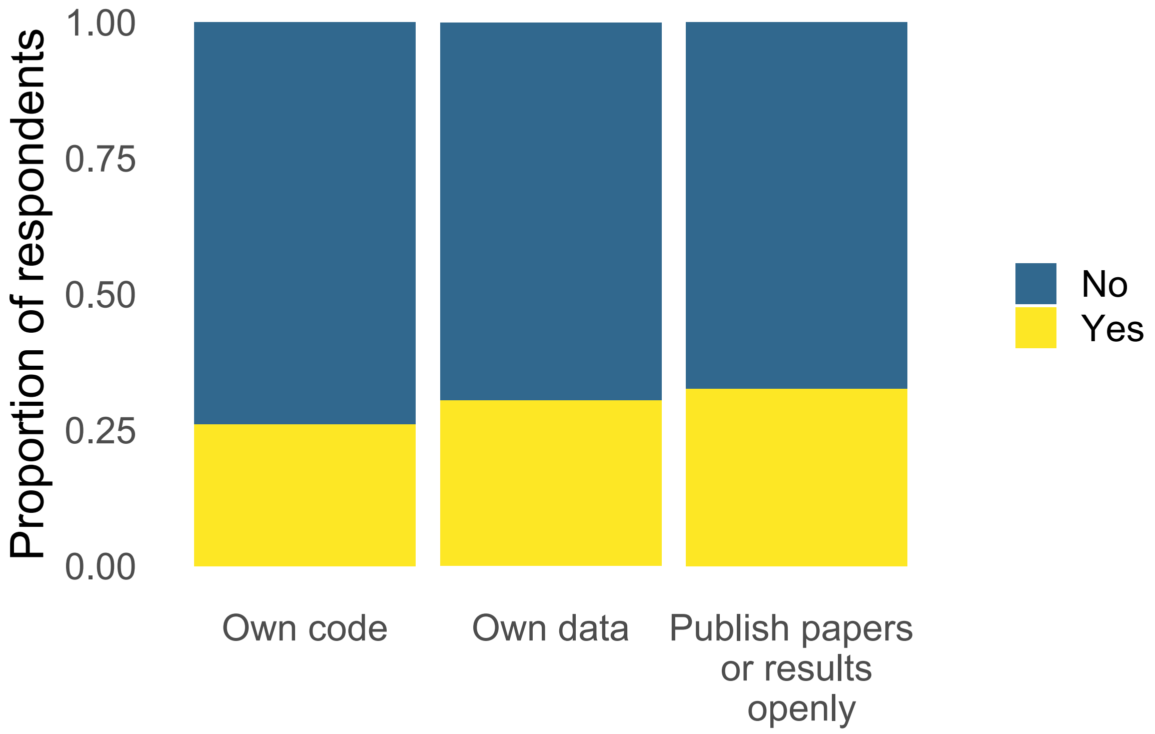

Figure 4: Respondents that had shared resources openly in own education.

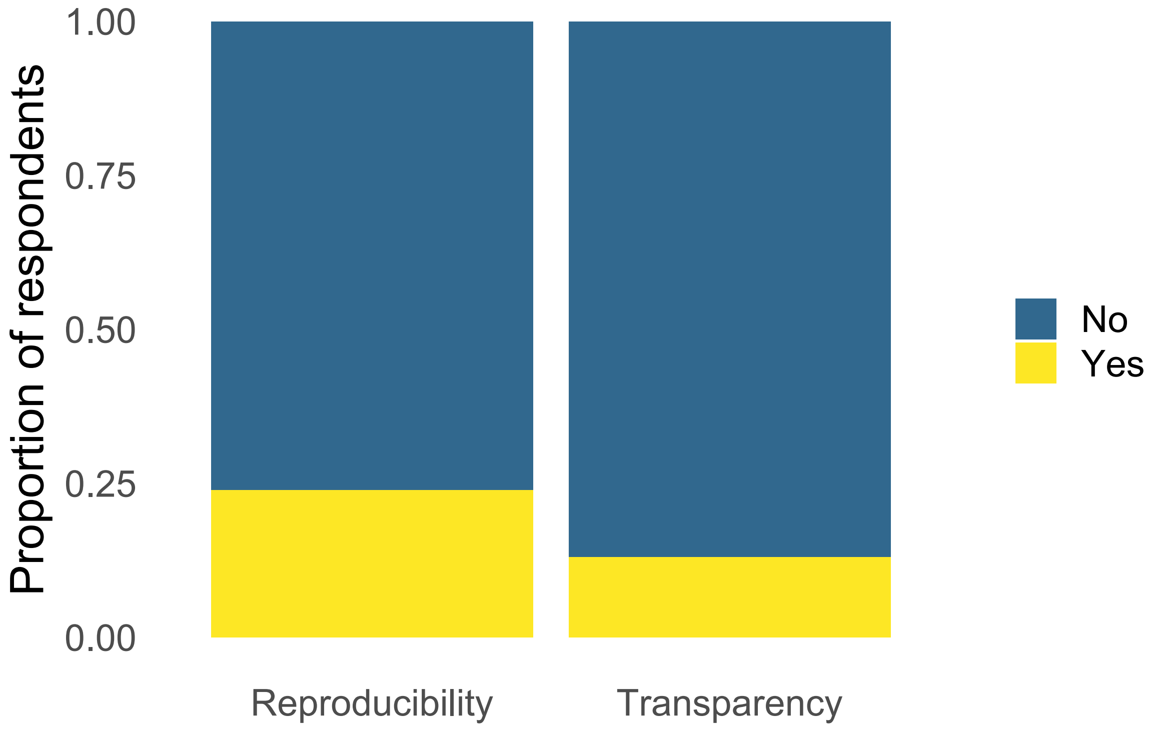

Figure 5: Respondents that had learned OS principles in own education.

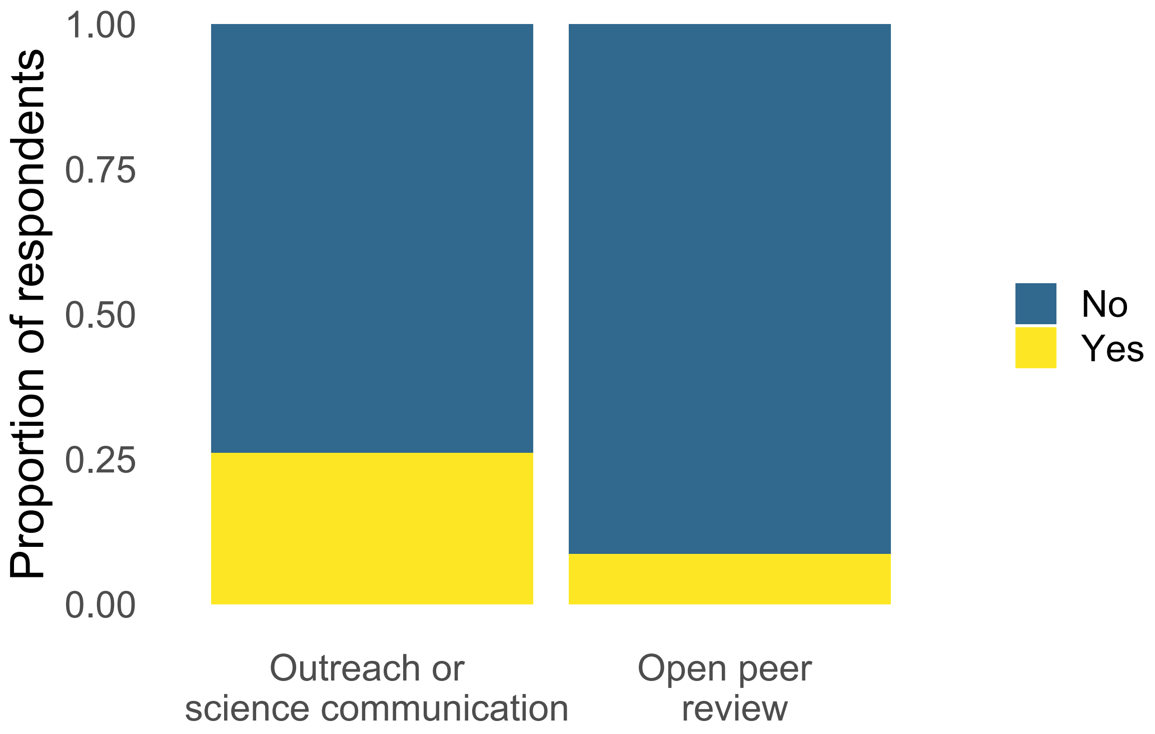

Figure 6: Open engamement among respondents in own learning.

Table 4: Codes assigned to participant responses during qualitative analysis of the "define open science" prompt. Note that many of the examples used could be, and ultimately were, assigned to two or more of the codes.

Code	Inclusion criteria and definitions	Examples
Transparency	Participant uses the words transparent, transparency or open to scrutiny.	Science based on data and methods that are completely transparent...
Accessibility/ Availability	Participant uses the words available or accessible or both in combination. Includes being understandable. Can reference being accessible to other scientists and/or to the public. Includes openly.	The sharing of scientific theory, data and results such that it can be accessed by anyone...
Sharing codes/ methods	Codes or methods are shared publicly and are available for others to use. Includes the idea that these methods should be free to access.	I define open science as a transparent process of doing Research, where the data, Methods, analyses and results are made available for others to replicate the study or analyse the data further.
Sharing data	Data is shared publicly and are available for others to use. Data re-use is included here. Includes the word interoperable outside of FAIR. Includes the idea that data sharing platforms should be free.	To have free, available data and papers. I think this work should be easily accessible to anyone. Data should not be altered (eg to hide information) and instead published with the intent of it being used by other researchers.
Working collaboratively with peers or other stakeholders	Open science facilitates collaboration or includes having stakeholders be a part of the research.	To me, Open Science means operating in a transparent manner. This could include making data and code available, facilitating research that might not involve yourself (which could be accomplished by making data and code available) and being open to working collaboratively to accomplish a common goal
Replication/ Reproducibility	Open science makes scientific findings replicable, reproducible, and/or verifiable.	...the methods used for analysing/interpreting the data are described/published in a way that makes them reproducible...
Open access publications	Publications are open access (i.e., not behind a paywall). May specifically refer to paywalls as a barrier. Includes pre-prints.	Scientific publications and the data used (can be anonymised) should easily without costs be accessible through internet.
FAIR principles	Specifically refers to FAIR principles: Findable, accessible, interoperable, and reproducible.	open sharing of data according to FAIR standards
Inclusivity	Indicates OS is inclusive and/or equitable; can refer to individuals or institutions and can reference making science possible for those who are not able to find funding.	Open science is research and knowledge that is available to everyone, regardless of social or economic situation.
Relationship between OS and Education	Participant discusses relationship between OS and education; however, may have views ranging from OS being part of education to the two concepts being in conflict.	In our education, we work on incorporating open science and training around data management and sharing for our students.
Data policies & practices	This includes metadata and following policies about data organization and documentation. Also includes standardization of sharing data.	It implies using standards for structuring and formatting data, standards for metadata, suitable web protocols, open source software and applications.
Responsible & available to the public	Specifically mentions that the public should have access to the data, methods, and or publications. Also, includes being responsible with public funds and making the information understandable to the public. Includes Citizen Science	When data is originally collected with public funding it should continue benefiting publicly funded research, and enhancing collaboration.
Other	Anything that is a one-off not included in the above or that is difficult to interpret. Includes open peer review, science done right, and proper credit. Includes that open science is fundable.	science made well

Table 5: Parameter estimates, SE and z-values for covariates in models used to test prediction 1.1

Fixed effect terms	Estimate	SE	z value
**global**			
University	0.76	0.43	1.79
YearLate	-0.38	0.44	-0.87
Men **	0.79	0.26	3.06
University:YearLate	0.03	0.54	0.05
**final**			
University **	0.82	0.27	3.02
Men **	0.74	0.26	2.84

Table 6: Parameter estimates, SE and z-values for covariates in models used to test prediction 1.3

Fixed effect terms	Estimate	SE	z value
**global**			
Use ***	1.05	0.26	4.08
Data	-0.47	0.25	-1.87
Men ***	1.43	0.42	3.41
**final**			
Use ***	1.04	0.26	4.03
Men ***	1.42	0.42	3.40

Table 7: Codes assigned to participant responses during qualitative analysis of the "what hinders your engagement in OS?" prompt. Note that many of the examples used could be, and ultimately were, assigned to two or more of the codes.

Code or category	Inclusion criteria and definitions	Examples
Cost	Lack of funding for publications or other funds needed to engage in OS.	Lack of funding to pay for fees in open access journals.
Insufficient incentives	There is no incentive to engage in OS from universities or funding agencies.	In the past years, there was little motivation to engage in Open Science.
Collaborators not using OS	When collaborators are wary or do not use OS, it makes it harder for them to use OS. This means they don't have opportunities to use OS.	Fellow scientists have been a bit skeptical about the idea of "giving away" their data.
Fear of critique	Afraid of people being overly critical of their work because it's available.	Fear of data being used to show that my work is wrong and flawed.
Legal concerns	Concern about either intellectual property, patent law, or data management law, such as GDPR not allowing them to share data.	My data is not mine but rather has been funded and collected by a country's regulatory agency. Otherwise I would freely make it available.
Insufficient knowledge	Don't know how to use OS. Afraid of trying OS platforms because they don't know how to use them.	Initially it was a lack of knowledge on how and where they could share code and data.
Time	Lack the time or it takes more time.	Lack of time. Work tasks and family life takes almost all available time.
More work	Engaging in OS takes more work. This is used when it's unclear that they are concerned about it taking more time, but specifically mention it taking more work or effort.	It requires extra work, setting up the data access and tidying up scripts and writing documentation.
Lack of guidelines	OS platforms or journals are unclear about how to upload data, data standards, meta data standards or other guidelines that make it difficult to know how to use them. Can also include if guidelines are regularly changing.	There are no clearly defined expectations, boundaries and mechanisms by which researchers can engage. Also it is never clear what precisely open science requires of me as a data contributor.
Want to get credit	Worried that OS practices, especially around data, will prevent them from getting proper credit for their work.	I can't speak from the experience but I would say fear of data being stolen or misused and no acknowledgement for the particular research.
Other/Vague	Unclear what they mean.	
Nothing	Specifically states that there is nothing hindering them.	

Table 8: Codes assigned to participant responses during qualitative analysis of the “what helps you to engage in OS?” prompt. Note that many of the examples used could be, and ultimately were, assigned to two or more of the codes.

Code or category	Inclusion criteria and definitions	Examples
Money	Having the money to engage such as money to pay for open publications.	Institutional funds for open access publications.
Resource availability	The existence of functional OS platforms, programs, or data that are available for use.	Online resources that are well written for relative beginners. User friendly platforms (e.g. OSF) that allow integration of multiple people from multiple institutions, and multiple types of files.
Social support	Encouragements or other support from their peers. This includes having a local or online community and the culture or movement around open science.	Working with people who are collaborative and like minded.
Having knowledge	They know how OS, including platforms or programs, works. Can include having taken classes/workshops. They have the necessary information to use these open resources. Implies a knowledge barrier that was overcome.	Increased information about options for open science.
Structural support	Programs or policies that support the adoption or use of OS that are provided by their department, institution, funding agency or journal requirements. This includes statements of encouragement from individuals in leadership positions. Institutional money for paying for publication costs goes under money not here.	Peer support and organisation’s own ICT department.
Intrinsic motivation	Reasons for engaging in open science that relate to positive or negative internal motivators such as wanting to be able to do particular types of experiments, feeling a need to pay it forward, their personal career development, or a personal philosophy that it’s the right thing to do.	Knowing that I have benefited from the resources provided by others and I have an obligation to ”pay it forward”.
Prior success with OS	Have had a positive past experience with OS that makes them more interested in continuing to engage in OS.	I have been able to access other people’s data and found it very useful in my career development hence a great motivation to do likewise.

Which OS aspects have practitioners encountered in their own formal education?

Table 9: Parameter estimates, SE and z-values for covariates in models used to test prediction 3.2

Model	Fixed effect terms	Estimate	SE	z value
**use data**				
global	use open data	0.97	0.61	1.58
global	Men	1.07	0.60	1.77
final	Men *	1.30	0.59	2.20
**use code**				
global	use open code *	1.38	0.60	2.30
global	Men	0.84	0.57	1.47
final	use open code *	1.31	0.59	2.21
**use open publication**				
global	use open publication	0.74	0.59	1.26
global	Men	0.15	0.58	0.26
**share data**				
global	share open data	1.11	0.59	1.88
global	Men *	1.50	0.60	2.51
final	Men *	1.41	0.59	2.40
**share code**				
global	share open code *	1.35	0.64	2.10
global	Men *	1.21	0.59	2.07
final	share open code *	1.45	0.64	2.27
**share open publicaiton**				
global	share open publication	1.33	0.74	1.80
global	Men	0.37	0.61	0.60
**use edu tools**				
global	use open edu tools *	1.85	0.73	2.51
global	Men	-0.22	0.60	-0.36
final	use open edu tools *	1.85	0.73	2.52
**do open review**				
global	do open review	2.16	1.11	1.95
global	Men	-0.30	0.60	-0.49
**do outreach**				
global	do open outreach	0.89	0.63	1.40
global	Men	-0.81	0.56	-1.44

Table 10: Parameter estimates, SE and z-values for covariates in models used to test prediction 3.3

Model	Fixed effect terms	Estimate	SE	z value
**global**				
global	Supervision	-0.17	0.27	-0.65
global	Teaching ***	-0.99	0.24	-4.14
global	Communication	-0.53	0.37	-1.44
global	Data	-0.40	0.37	-1.07
global	Method	0.02	0.38	0.05
global	Publish	-0.56	0.38	-1.45
global	Reproducibility	0.69	0.39	1.76
global	Transparency	0.69	0.39	1.77
global	Men	0.77	0.60	1.30
**final**				
final	Supervision	-0.16	0.27	-0.59
final	Teaching ***	-0.99	0.24	-4.13
final	Communication	-0.53	0.37	-1.44
final	Data	-0.40	0.37	-1.07
final	Method	0.02	0.38	0.05
final	Publish	-0.56	0.38	-1.46
final	Reproducibility	0.68	0.39	1.75
final	Transparency	0.69	0.39	1.76

FIRST PAGE: CONSENT

About the study

This is the first step of a three-part survey of the participants of the Living Norway 2020 Colloquium. The aim of the survey is to investigate perceptions of open science, open science applications, as well as conditions that either promote or limit open science practices. Further, we are interested in understanding how perceptions of and attitudes towards open science develop during the colloquium, as well as the participants' feedback on the Living Norway Colloquium itself.

This will be done through three linked surveys sent out before, during, and just after the colloquium.

To link the three survey parts together at the end of the workshop, we ask you to provide your email address as a temporary identifier for this purpose only. Once the surveys are linked, the email addresses will be immediately and permanently removed from the survey dataset and survey participants will be unidentifiable.

The University of Bergen (UiB) is responsible for conducting the survey and will ensure that all submissions are handled confidentially. Data will be stored on a safe server and will only be accessible to researchers responsible for summarizing the survey data on behalf of Living Norway. None of the respondents will be identifiable in the final data or in any communication of the results from the study.

The surveys are important to us in developing Living Norway Colloquia and in better understanding the research community's views and opinions of open science issues. As this study requires the full consent of participants, you are free to withdraw at any time without the need to justify.

The study is registered in the UiB data and privacy framework RETTE.

Your rights

As long as you are identifiable in the collected data, you have the right to the following:

- View of your registered personal data.
- Correction of your registered personal data.
- Deletion of your registered personal data.
- Access to a copy of your registered personal data.
- File a complaint for the UiB privacy agent (persornvernombud) or the Norwegian Data Protection Authority (Datatilsynet) regarding the handling of your registered personal data.

If you have any questions about the study, please contact

- Professor Vigdis Vandvik, bioCEED/UiB, vigdis.vandvik@uib.no
- Christian Bianchi Strømme, bioCEED/UiB, christian.stromme@uib.no

###Consent

By answering the following survey questions, I confirm that I have received information about the study and that my participation is voluntary.

In order to link your answers, we kindly ask you to provide your email address in the field below. Please use the same address on all parts of the survey:
