# Supplementary material for: Close to open—Factors that hinder and promote open science in ecology research and education
Source: PLoS One. 2022 Dec 21;17(12):e0278339. doi: 10.1371/journal.pone.0278339 (PMC9770360; doi:10.1371/journal.pone.0278339)
Supplement: Attachment — Submitted filename: Response to reviewers.docx [file pone.0278339.s002.docx]

**Response to reviewers**

Dear reviewers,

We are truly grateful for the sensible comments that you provided in your review of our first version of the manuscript. This input have been very helpful to our efforts in making an improved version. You will find your comments addressed point-by-point below with line numbering referring to lines in the new version.

We also expanded our efforts to perform additional improvements. These were mainly made with the purpose of clarifying structure and content in the discussion by referencing our results more explicitly as a basis for the broader contextualisation of findings there. Further, we made minor changes throughout the manuscript to improve consistency in the use of terminology as well as clarifying language. Finally, we also took the opportunity to reformulate the title to increase appeal to readers.

We hope that you have the opportunity to assess this new version.

Yours sincerely,

On behalf of the authors,

Dr. Christian Bianchi Strømme

Reviewer #1:

*1.1 Recommend adding the Human Subjects IRB Statement in the Ethics statement part of the application in the actual manual script under the methods section so those who want to look up the protocol can.*

Thank you for indicating the lack of this type of information. We have articulated how we informed respondents of their rights, how we asked for their consent and how their privacy was protected in how we managed data (lines 94-97). In addition, we added content on how we conferred with the Data Protection Officer of the Norwegian research data authority for data management as well as where the project was registered according to the agreed procedure (lines 104-107).

*1.2 Recommend authors explain how they came up with their preregistered predictions (rows 127-136)*

In compliance with your kind advice, we have added rationales for each of the formulated predictions A-E on lines 141-14, 148-151, 153-156, 158-160 and 163-165, respectively.

*1.2.1 Some of the predictions make sense but not all. The second bullet in particular is not super obvious on why colloquium participants would use open data more than contributing to open data and code—is it the colloquium participants or is it that it’s typically easier for people to be users of open data rather than creators?*

Thank you for indicating this need for clarification. For this prediction, we explicitly refer to the colloquium participants since these constitute the surveyed population in the study. Further, we expected that these would be more frequent users of open data rather than contributors, both due to the effort required in generating data vs. using available data, as well as existing incentives for publishing as compared to fewer incentives for sharing data.

*1.3 Recommend moving some or most of the information presented in the aimsandhypotheses.Rmd file on Github to the main text of the manuscript. There are a lot of assumptions in that file that is not clear in the manuscript and for those who are less inclined or not familiar with Github, they’re going to be missing crucial elements of what the predictions are or how the predictions came to be. Explanations for why the authors are assuming each of the predictions are needed.*

We agree with this assessment. As indicated for 1.2, this information is now included in the main body of the revised version by adding the rationales for each of the formulated predictions (lines 139-165). We have also added further content on how we came up with these predictions (lines 130-134).

*1.3.1 For prediction 1.1: the 4 items that are listed (academic affiliation, year of degree, frequency of engagement with open science practices, and gender) are, I am assuming, the items that would be used in the model. But as the prediction is currently written (researchers with academic affiliation have engaged more in open science-related practices compared to other researchers, and more so for early-career researchers), there is no topic for non-academic affiliation. 1.1 is stated that those in academia will have more engagement with open science practices than those not in academia, which I’m assuming to mean research institutions, think tanks, non-profits, industry, etc. It might be a mis-labeling but “academic affiliation” would not capture those at non-academic institutions. Suggest changing from academic affiliation to just “type of affiliation.”*

We agree that this prediction required more precise wording, and we have thus phrased it accordingly. The justification for this prediction is that we expected researchers affiliated with universities to engage more frequently with open science when compared to other types of institutions because of stronger incentives here and because many research institutions have and manage proprietary data.

*1.3.2 Prediction 1.3: similar to above, why is the assumption that using open data is more likely than contributing? There’s no explanation for this assumption. Again, maybe just needs to be rephrased—the research question could be something like “Do workshop participants more frequently use or contribute to open data and code?”*

Thank you for pointing towards the lack of information, and the formulated rationale for this prediction is at lines 148-151.

*1.3.3 Similarly, prediction 1.4: this is an assumption but there’s no explanation for why the authors assumed this.*

We agree that also this prediction required a justification. The rationale for this prediction is added at lines 153-155.

*1.3.4 Similarly, prediction 3.3: an explanation for why would be helpful.*

As for the preceding prediction, we added a rationale for this prediction, here based on the incentives for research compared to those for teaching and supervision (lines 163-165).

*1.4 Row 142: extra “ ).” after “package Ordinal[12].” at the beginning of the row.*

Thank you for identifying this error, it is now corrected (line 172).

*1.5 Row 329: methods is misspelled as “metohds”*
Thank you for identifying this, we have corrected this error (line 355)

1.6 Row 334: extra “m” in large
Thank you for pointing out this error. Please note, the phrase is no longer present in the revised version.

*1.7 The results headings don’t match the things called out by the authors. It was a little difficult to go back and match what the research questions were relative to what predictions were made. Recommend changing either the predictions to be research questions in the methods section or the headings to match the predictions in the results section for ease of going back and forth for the reader.*

By the help of your input on this aspect, we acknowledged that the previous version of the manuscript did not have a sufficiently clear description of the study design. We applied a mixed-methods approach to our study questions by combining two separate lines of inquiry that used qualitative and quantitative analyses, respectively, of the data that we gathered. This is now stated explicitly in the paragraph were the study questions are expressed (lines 56-58), while also referred to in the subsection “Survey structure and content” (lines 90-91) and in the first paragraph of the discussion section (lines 326-327). We did this for making clear that the predictions were embedded in the quantitative line of inquiry, and thus subservient to the research questions that are mirrored in the subsections in the results section.

*1.7.1 Some of the headings don’t appear as part of the authors’ predictions at all, which is fine but it throws off the format and what the reader is expecting. For instance, “What are the perceived benefits and risks for individual engagement in OS?” is a survey question that got its own write-up but there were no predictions on this.*

For this study question, we did not formulate quantitative or scaled survey items since we did not want to influence or impose perceptions of benefits or risks. Instead, we used two open-ended survey items (one for helps and one for hinders to OS engagement) to capture the ideas of respondents on those topics. We hope that the added content referenced above help clarify this aspect.

*1.7.2 It might also be helpful to frame the discussion based on the authors’ predictions for reading ease as well.*

Being granted the possibility to submit an improved ms on our study, we (the group of authors) agreed on the need to clarify content in the discussion by referencing our results more explicitly as a basis for the discussion. We have done so in the first (lines 324-341) and second (lines 342-350) paragraphs of the discussion. In our interpretation of the results on the perception of OS, how respondents statedly engaged with OS and what they saw as influencing those actions, we see a range of potentials for supporting mechanisms that can help promote implementations of OS in research and society. Thus, paragraphs 3-6 are thematically and sequentially structured to guide such efforts on the basis of referenced guidance in the literature and our results where relevant. For the purpose of clarifying structure and content, we have taken the opportunity to reduce content down to seven paragraphs in the discussion.

*1.8 The authors looked how practitioners interact with and the frequency of their interaction with OS by gender and found that OS practices was higher for men than for women. This finding isn’t discussed at all—what does it mean for men to have more frequent interactions in OS than women? Is this important? Is this disparity something that needs to be addressed? The authors must have thought there was some importance since they put it as part of their predictions. Why do men interact more with OS than women? It would be great if these could be addressed in the discussion somewhere.*

Since the inclusion of the Gender-term in the quantitative analyses was exploratory in nature, we have added a statement on this in the paragraph after the predictions (lines 167-168). Following your suggestion, we have included a discussion of these findings at lines 406-411),

*1.9 The authors touch on the fact that this is a pro-OS group in general as they are participants of Living Norway, a workshop dedicated to openness and transparency. The study shows that even among people who recognize the importance of OS, most are participating in OS by using open code and open data and reading open access publications—all very passive forms of participating in OS. This suggests quite a large problem—if those who are biased towards OS aren’t active creators of OS, then what does this mean for those who don’t believe in the practice of OS to begin with? The recommendations made in the discussion to include OS as part of the teaching curriculum stems from the results the authors found but it doesn’t touch on the cultural aspect that there’s just not a big push for people to be more active in their OS practices. It would be great if the authors could address how to get OS teaching into the curriculum by faculty researchers and institutions who may not value OS as much as those participating in the Living Norway workshop. Workshop and survey participants are already the best case scenario in that they’re proponents of OS, the recommendations match and are in line for these types of individuals. The authors should address this bias and provide other ways for OS to be integrated among groups or individuals who don’t perceive OS to be as important or useful.*

Thank you for elaborating on this aspect in your advice, we realised that we did not address this bias in the previous version. Following the paragraph on where and how educators can find recommendations and guidance both in general and specific terms (lines 351-370), we have both reformulated previous content and added new content on the role of institutions and policy-makers in facilitating OS in higher education (lines 371-382). Along these lines, we have also clarified and expanded on some of the potential caveats that can accompany efforts for introducing OS in higher education in the second-last paragraph (lines 401-417).

Reviewer #2:

*2.1 Line 28-29 – I would be tempted to change “sub-optimal piecemeal manner”, to something softer, potentially drawing on language used in the discussion. Maybe something like “unified/uncoordinated/disconnected grassroots efforts”. I really like the discussion point about bridging multiple levels of OS practices to build a fully OS adopted science, and that cooperative language might be nice to sprinkle throughout the MS.*

Thank you for indicating this, we agree on the necessity of improving this sentence and have reformulated accordingly (lines 28-31).

2.2 Line 104-120 – I appreciated the detailed description of the coding.

Thank you.

*2.3 Line 125 – Would it possible to link to the specific file (*[*https://github.com/christianstromme/LivingNorway2020/blob/main/Research%20hypotheses/aimsandhypotheses.Rmd*](https://github.com/christianstromme/LivingNorway2020/blob/main/Research%20hypotheses/aimsandhypotheses.Rmd)*), just for ease of navigating the GitHub repo. This partly links back to my comment on the usage of drake. Superbly tidy GitHub repo, just not immediately accessible for everyone and perhaps a little more specifies for some files could help.*

We have modified accordingly, it is much better to link the file directly as you kindly indicated.

*2.4Line 137 – Would it possible to add the R version alongside this citation. On another note, I would be tempted to cite all the R packages used. I feel this could be done via an additional supplementary table if you feel it would break the flow of the MS proper. The grateful package (*[*https://github.com/Pakillo/grateful*](https://github.com/Pakillo/grateful)*) might be a quick way of sorting something like this out.
Table 1 – might be tempted to add % of sample to each value as part I and II have different sample sizes (which is already nicely highlighted in the table headings).*

Thank you, we have now added the R version to the citation accordingly.

*2.5 Line 307-309 – I think summarises the findings nicely, and ties nicely in with the next paragraph. People have a steady idea of OS, but the formalisation is required to make it more widespread.*

In our efforts to clarify content in the new version, we have rephrased this content slightly to reference our results more broadly (lines 336-338). Further, we follow with content also referencing the lower perceived importance of OS in teaching compared to research (lines 338-341).

*2.6 Line 333-335 – Very clear limitation on the generalisability. Doesn’t undermine how interesting the MS is, and I very much appreciated its inclusion in the discussion.*

Although we removed this sentence as part of our efforts to clarify content in the discussion, we have simultaneously made sure to consistently limiting our interpretations to the given context and to the extent to which the qualitative and qualitative lines of inquiry in our design can inform each other.

*2.7 Line 352-356 – really like the connection built here and the highlighting of increased facilitation could help OS, in particular the mention of incentives.*

In addition, we have concluded that paragraph by summarising the forms of support that can help promote OS.

*2.8Line 372-373 – I agree that OS could be perceived as more for advanced students. But equally I think there may be value in highlighting how OS practices could be connected to teaching on ethics and researcher integrity. Undergrads are taught citational practices, expanding that teaching to include data citation and learning the benefits of open data seems like it would fit nicely.
I wonder whether the early the better if it can be tied to researcher integrity aspects of undergrad teaching. I think you’ve largely captured what I am trying to say in the paragraph on line 380-389, but I would be tempted to squeeze in “researcher integrity” to capture that buzzword. I really liked how inclusivity was brought up in the results, and potentially could be re-highlighted in the discussion with this concept of researcher integrity. Paired with participants comments about “fear” I think you could make a strong point about attitudes, rooted in inclusivity, could boost OS uptake. There is a very practical point on line 386 of boosting careers, but I think equally valuable is promoting researchers etc that are a pleasure to work with.*

We agree that the passage you referenced in your review of the first manuscript would have been an appropriate section to include further guidance on how OS can be included in teaching by building on researcher ethics. Since we have shortened the discussion and modified the structure somewhat in the new version, we have instead elaborated further on the third paragraph of the discussion (lines 351-370) where we reference examples and guidance for introducing OS in teaching and learning.

2.9 Line 432 – Great ending line.

Thank you.
