# Supplementary material for: Close to open—Factors that hinder and promote open science in ecology research and education
Source: PLoS One. 2022 Dec 21;17(12):e0278339. doi: 10.1371/journal.pone.0278339 (PMC9770360; doi:10.1371/journal.pone.0278339)
Supplement: Attachment — Submitted filename: Response to the reviewers.docx [file pone.0278339.s003.docx]

**REPLY TO THE REVIEWERS**

We are very grateful for the helpful comments, as they pointed to needed edits in the previously submitted manuscript. Please find the point-by-point replies and additional edits with line references below.

*Reviewer #1*

1) We agree with Reviewer 1 that this is an interesting aspect, as it can potentially impact institutional support for open science (OS). Though such impacts would ultimately manifest on the level of individual actions and motivations, we did not survey perceptions of research assessment or its connection to OS in explicit terms through the survey we developed. Further, our concern is that including this aspect post-hoc would be too speculative, especially since we did not identify the idea of research assessment across the responses to the survey items on helping and hindering individual OS engagement. Even so, we agree on the need to better describe the EU scenario in this respect. We have added a statement on the prevailing emphasis of research papers and external funding for individual careers as indicated by findings from a Europe-wide survey in 2019 (lines 385-388, reference at line 534). Thus, the passage on the Utrecht University that now follows the added sentence is an example of how research assessment can be adjusted for promoting OS.
2) Agreed. We have moved website references (found at lines 17, 47, 52, 134, 359 and 360) to the reference list (corresponding lines 447, 450, 477, 480, 486, 507 and 510) as suggested.

*Additional changes*

Line 388: We replaced “Even so” with “Meanwhile” to increase flow between the anteceding (newly inserted) sentence and this sentence.
